# Anatomical variation in the ankle and foot: from incidental finding to inductor of pathology. Part I: ankle and hindfoot

**DOI:** 10.1186/s13244-019-0746-2

**Published:** 2019-07-31

**Authors:** Maria Pilar Aparisi Gómez, Francisco Aparisi, Alessandra Bartoloni, Maria Alejandra Ferrando Fons, Giuseppe Battista, Giuseppe Guglielmi, Alberto Bazzocchi

**Affiliations:** 1 Department of Radiology, Auckland City Hospital - Auckland District Health Board (ADHB), 2 Park Road, Grafton, Auckland, 1023 New Zealand; 2 Department of Radiology, Hospital Vithas Nueve de Octubre, Calle Valle de la Ballestera, 59, 46015 Valencia, Spain; 3 Department of Diagnostic Imaging, Bambino Gesù Children Hospital, Piazza Sant’Onofrio 4, 00165 Rome, Italy; 4 Department of Orthopaedics and Traumatology, Malteser Krankenhaus St. Josefshospital, Kurfürstenstrasse 69, 47829 Krefeld, Germany; 5 Department of Experimental, Diagnostic and Specialty Medicine (DIMES), University of Bologna, S.Orsola-Malpighi Hospital, Via G. Massarenti 9, 40138 Bologna, Italy; 6 Department of Radiology, University of Foggia, Viale Luigi Pinto 1, 71100 Foggia, Italy; 7 Diagnostic and Interventional Radiology, IRCCS Istituto Ortopedico Rizzoli, Via G. C. Pupilli 1, 40136 Bologna, Italy

**Keywords:** Ankle, Accessory ossicles, Accessory muscles, Computed tomography, Magnetic resonance

## Abstract

Accessory anatomical structures in the ankle and foot usually represent incidental imaging findings; however, they may also eventually represent a source of pathology, such as painful syndromes, degenerative changes, be the subject of overuse and trauma or appear as masses and cause compression syndromes or impingement.

This review aims to describe and illustrate the imaging findings related to the presence of accessory ossicles and muscles in the ankle and hindfoot through different techniques, with special attention to those variants that associate factors of clinical relevance or that trigger challenges in the differential diagnosis.

## Key points

- Accessory anatomical structures in the ankle and hindfoot are a common incidental finding.
- Anatomical variants may trigger challenges in the differential diagnosis
- Anatomical variants may be a source of pathology

## Introduction

A number of anatomical variations can be found in the ankle and hindfoot. These include accessory ossicles, additional sesamoid bones, variations in number and configuration of sesamoid bones, coalitions, bipartitions and variants in the soft tissues, such as accessory muscles.

Most of them represent developmental abnormalities that constitute incidental radiographic findings [1].

Accessory ossicles in most cases are a result of unfused ossification centres. They are seen as subdivisions of existing bones or free elements in the vicinity of the normal bone structures. Sesamoid bones have a different anatomical nature. They functionally represent components of a gliding mechanism and are at least partially embedded in tendons, reducing friction and protecting the tendon structure [1, 2].

The most common accessory ossicles in the ankle and foot are the os trigonum, the accessory navicular (among the different three types, type II is the most common) and the os intermetatarseum, in this order. With respect to accessory sesamoid bones, the os peroneum is the most frequently found [2].

Accessory muscles are also generally asymptomatic and discovered incidentally on imaging studies. Occasionally, they will manifest clinically, presenting as mass lesions or causing compression syndromes such as tarsal tunnel syndrome, chronic pain or impingement.

Our aim with this review is to illustrate the imaging findings related to the presence of accessory ossicles and muscles in the ankle and hindfoot through different techniques, with special attention to those variants that associate factors of clinical relevance or, in the case of the ossicles, that would pose a challenge in the differential with fractures.

Bone coalitions, given their complexity and frequent clinical implications, deserve separate analysis and will not be the object of this review.

## Ankle and hindfoot

**Fig. 1:**
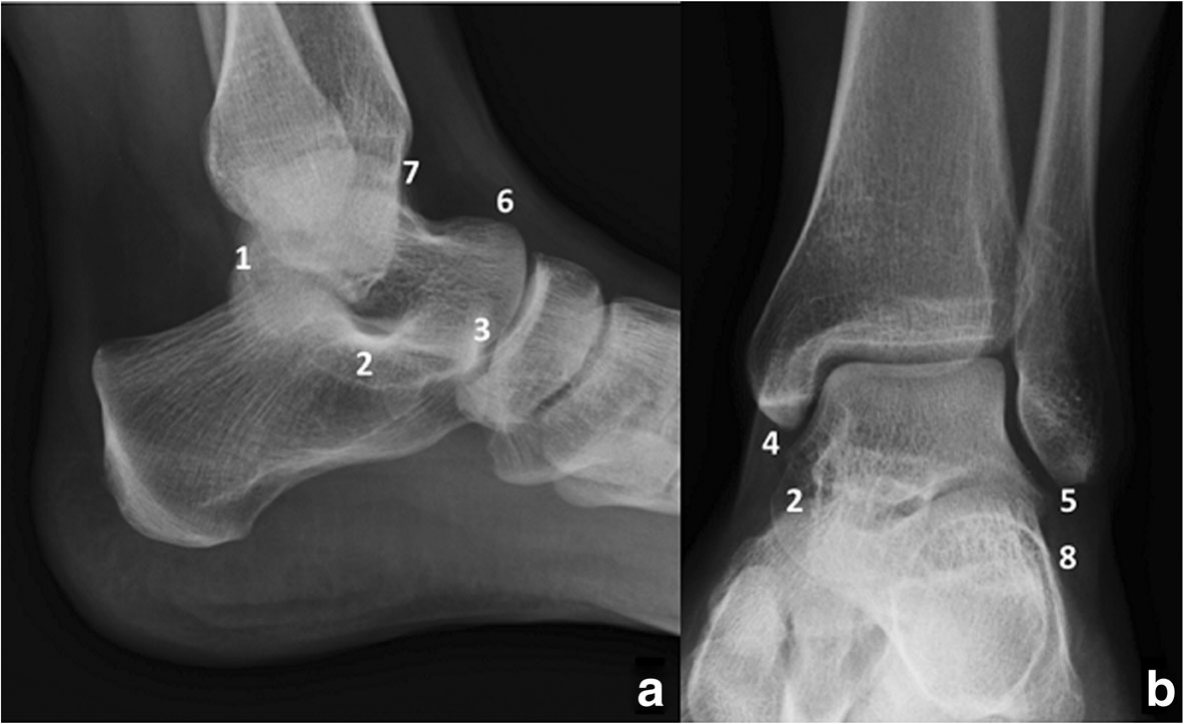
Diagram of the location of the most common accessory bones around the ankle and hindfoot. **a** Lateral and (**b**) AP projection of the ankle and hindfoot: 1—os trigonum, 2—os sustentaculi, 3—os calcaneus secundarius, 4—os subtibiale, 5—os subfibulare, 6—os supratalare, 7—os talotibiale, 8—talus secundarius

- Ossicles (Fig. 1) (Table 1)

**Table 1:** Prevalence, clinical significance and differential diagnosis of the most common types of accessory ossicles around the ankle and hindfoot

Ossicle		Prevalence	Clinical significance	Differential diagnosis
Os trigonum		1–25%	Os trigonum syndrome	Posterior ankle impingement, Cedell’s fracture, Shepherd’s fracture
Os sustentaculi		0.3–0.4%	Coexisting talo-calcaneal coalition	Fractures of the sustentaculum tali
Os calcaneus secundarius		0.6–7%	–	Fracture of the anterosuperior calcaneal process
Os subtibiale		0.9%	–	Avulsion fractures
Os subfibulare		2.1%	–	Avulsion fractures
Rare	Os supratalare	–	–	Avulsion fractures
	Os talotibiale			

### Os trigonum

The os trigonum is one of the most common accessory ossicles in the ankle and foot. Its prevalence is estimated in between 1 and 25% [1, 2].

Initially, this was interpreted as a non-united fracture. Currently, this is viewed as a developmental skeletal variation, likely resulting from failure of fusion of a secondary lateral tubercle ossification centre that forms at about 7–13 years of age, and normally fuses at about 14 years of age [1, 3–5]. It is connected to the lateral tubercle of the posterior process of the talus by a fibrocartilaginous synchondrosis and in close vicinity to the flexor hallucis longus tendon [6].

The os trigonum is normally an incidental finding, with no associated pathology. Os trigoni may have a round, oval or triangular morphology [6].

**Fig. 2:**
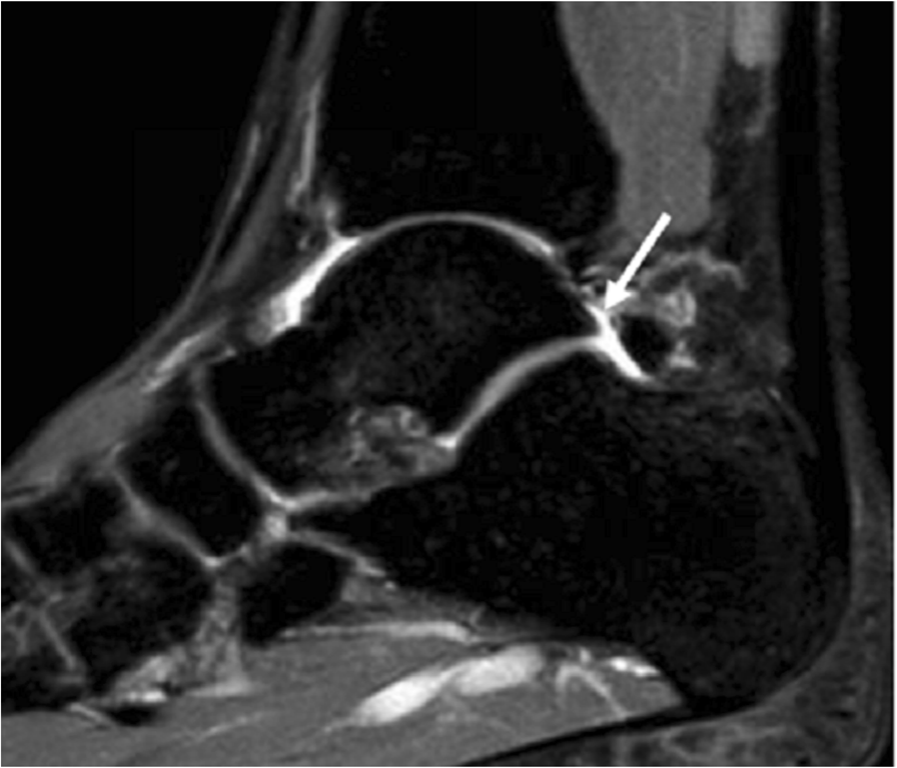
24-year-old man, history of ankle sprain and persisting pain in the lateral and posterior aspect of the ankle. Sagittal proton density spectral attenuation inversion recovery (PD SPAIR) image. Note the hyperintense band of fluid between the os trigonum and the posterior aspect of the talus, in keeping with disruption of the synchondrosis (white arrow). Injury to the synchondrosis is one of the causes of symptomatic os trigonum

A single traumatic episode of forced plantar flexion or repetitive forced plantar flexion may result in degeneration or tear of the synchondrosis (Fig. 2). Repetitive plantar flexion is a continuous requirement in activities such as ballet, basketball or soccer [5, 7]. In these cases of repetitive trauma, there can be involvement of the soft tissues, which results in irritation manifested as local synovitis, flexor hallucis longus (FHL) tenosynovitis or entrapment.

Symptoms of os trigonum syndrome may result from all the situations mentioned above and consist of chronic or recurrent pain with stiffness, soft tissue swelling and tenderness to palpation in the postero-lateral aspect [8]. The os trigonum syndrome constitutes a subtype of posterior ankle impingement syndrome [2, 5].

Radiographs can detect the presence of an os trigonum; however, their sensitivity is limited in the assessment of early bone changes occurring with the development of pathology. The posterior impingement view has been shown to be more sensitive than the lateral view in the detection of an os trigonum [9].

Computed tomography (CT) and magnetic resonance imaging (MRI) are able to detect the associated bone and soft tissue abnormalities.

On CT images, irregularity and sclerosis, or changes related to degenerative change on the articular surfaces of the synchondrosis, may be present as a result of chronic stress and abnormal movement [10].

**Fig. 3:**
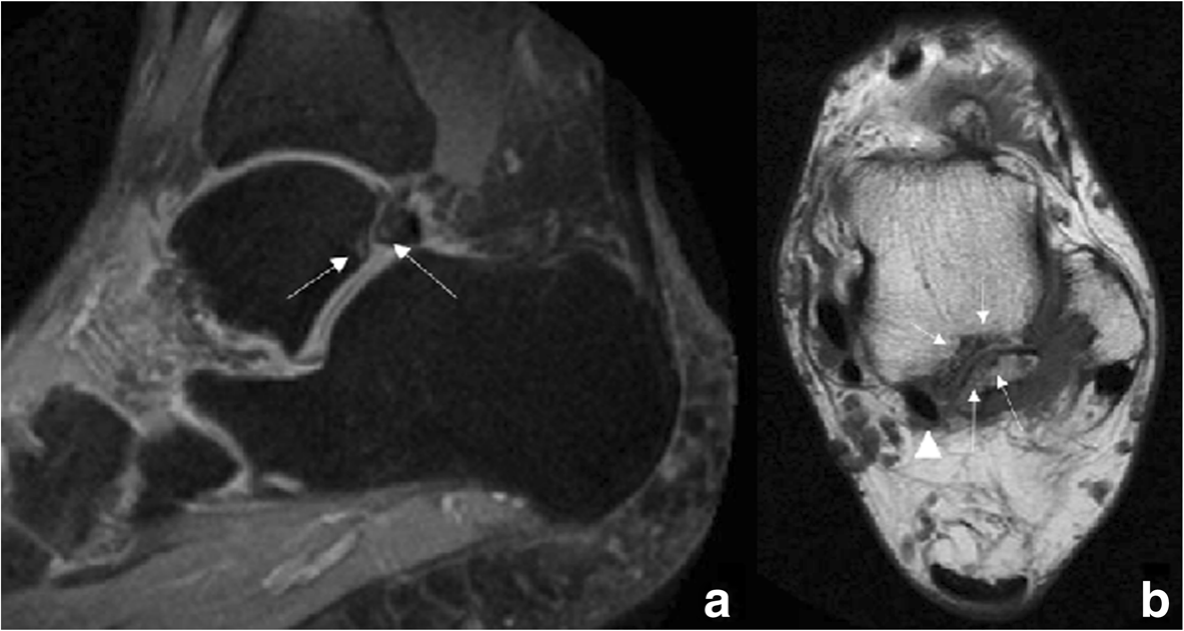
52-year-old man with persisting posterior ankle pain. **a** Sagittal fast spin-echo proton density (FSE PD) fat sat demonstrates an os trigonum. There are foci of increased signal intensity in the subchondral bone in both aspects of the synchondrosis (white arrows), with a small amount of fluid in the posterior aspect of the tibio-talar joint. **b** Axial fast spin-echo T1 (FSE T1) better depicts the presence of foci of subchondral bone oedema and subchondral bone cysts in both aspects of the synchondrosis (white arrows). Note how the os trigonum is intimately related to the flexor hallucis longus (arrowhead)

On MRI images, the presence of bone marrow oedema in both aspects of the synchondrosis, fluid within or joint effusion, or signs of soft tissue involvement, such as oedema in surrounding structures or tenosynovitis of the flexor hallucis longus, will be indicative of os trigonum syndrome [6] (Fig. 3).

Increased technetium-99m intake is another typical feature linking symptoms to the presence of an os trigonum [2], although bone scans are currently not so widely used due to the increased use of MRI. Fluoroscopically guided injection of lidocaine into the synchondrosis was used as a diagnostic test in the past to accurately determine the cause for symptoms [5, 6].

**Fig. 4:**
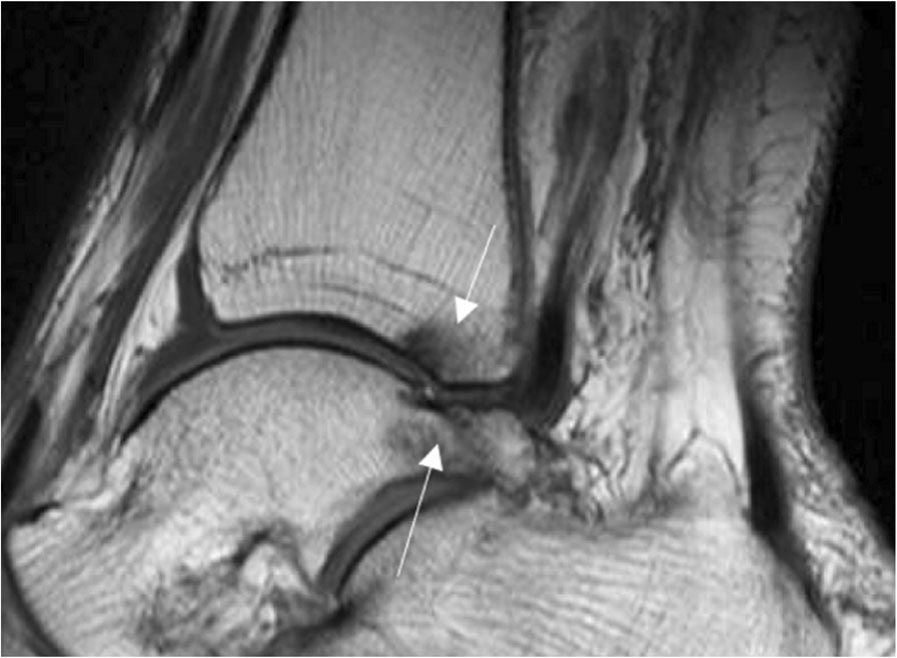
Stieda’s process. 66-year-old woman, presented with pain in the posterior and lateral ankle. Sagittal FSE T1 demonstrates an elongated postero-lateral process of the talus (Stieda’s process). There is subtle subchondral bone marrow oedema in the process and in the posterior aspect of the distal tibia (thin white arrows), indicative of mechanical overload

Other possible causes of posterior impingement are related to the morphology of the lateral tubercle of posterior process of the talus (also called Stieda process). When this is markedly prominent, the same mechanism of plantar flexion described above [2, 11] causes similar pathology in the surrounding anatomical elements (Fig. 4).

The differential diagnosis of an os trigonum comprises fractures of the lateral or medial tubercles of the posterior process of the talus. These represent Shepherd’s or Cedell’s fractures, respectively [9, 12]. These fractures result from acute impingement between the posterior aspect of the tibia and the calcaneus on extreme flexion of the ankle, with damage to the interposed posterior process of the talus. In the cases of Shepherd’s fracture, there will be postero-lateral tenderness with pain on movement of the subtalar joint and with passive movement of the flexor hallucis longus.

In the case of Cedell’s fracture, pain will be located postero-medially and, in some occasions, a lump will be palpated [13, 14].

Fragments are usually non-displaced and resemble os trigonum. In the cases of fracture, besides from the background of trauma that may suggest it, on CT or MRI assessment, the edges will be irregular, not corticated and comminution is possible [2]. Fractures of an os trigonum itself are extremely rare [8].

These fractures can sometimes be missed, and they need fixation; otherwise, they may result in pseudoarthrosis. If fragments are very small, they can be excised. In cases when non-union has occurred, the fragments are usually resected [14].

### Os sustentaculi

The os sustentaculi represents a very rare skeletal variant of the ankle and foot region, with a prevalence that has been estimated in 0.3–0.4% [1, 3]. This is connected to the posterior aspect of the sustentaculum tali by a fibrocartilaginous synchondrosis.

**Fig. 5:**
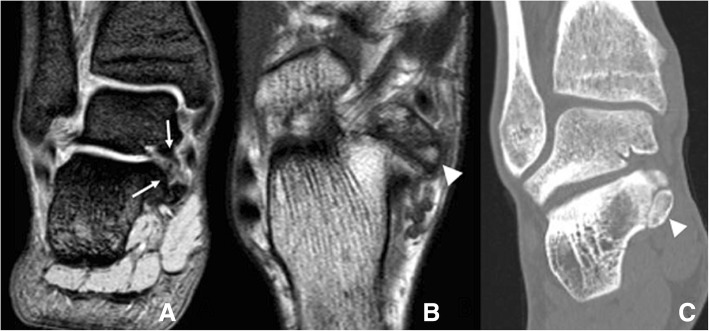
Os sustentaculi. **a** 17-year-old man referred with the suspicion of peroneal tenosynovitis. **a** Coronal T2-weighted fast field echo (FFE) demonstrates a talocalcaneal coalition (white arrows). **b** On axial FSE T1, a small ossicle is visible, interposed in between the two coalescent bones, in keeping with an os sustentaculi (arrowhead). **c** Reconstructed coronal CT image in the same patient, demonstrating the os sustentaculi (arrowhead), in close relation with the sustentaculum tali

A variant of the os has been called the assimilated os sustentaculi. This represents an accessory joint between the bony projections at the sustentaculum tali and the adjacent talus. It has been speculated that the os may incorporate to the sustentaculum when growth finishes, and this represents a fused variant of the condition [15, 16]. A talocalcaneal bony bridge at the posterior aspect of the sustentaculum can represent fusion of the os with both bones, calcaneum and talus, effectively constituting a subtalar coalition (Fig. 5).

In a recent retrospective study by Yun et al., it was found that coexisting intraarticular talocalcaneal coalition was observed in 11 of 13 patients with extraarticular talocalcaneal coalition with os sustentaculi, suggesting that the os sustentaculi is a component of extraarticular talocalcaneal coalitions and thus related to the presence of symptoms [17].

This os can become symptomatic in cases of chronic abnormal mobility (shearing), in which degenerative changes across the synchondrosis occur. Typical findings on CT images will be irregularity of the synchondrosis surfaces, with sclerosis and subchondral cyst formation. On MRI, there will be subchondral bone marrow oedema and fluid [15].

Differential diagnosis includes fractures of the sustentaculum tali, which are also rare. These occur when there is a direct impact on a supinated foot and are frequently seen in association with severely comminute intraarticular calcaneal fractures, although they can also be found isolated. Pain and tenderness along the medial aspect of the foot will be the main clinical symptoms [13].

Besides from the background of trauma, typical features of a fracture such as irregular interfaces and no cortication help to establish the diagnosis on radiographs and CT. Associated bone marrow and soft tissue oedema typically associated to the presence of fractures will be seen on MRI [2].

### Os calcaneus secundarius

**Fig. 6:**
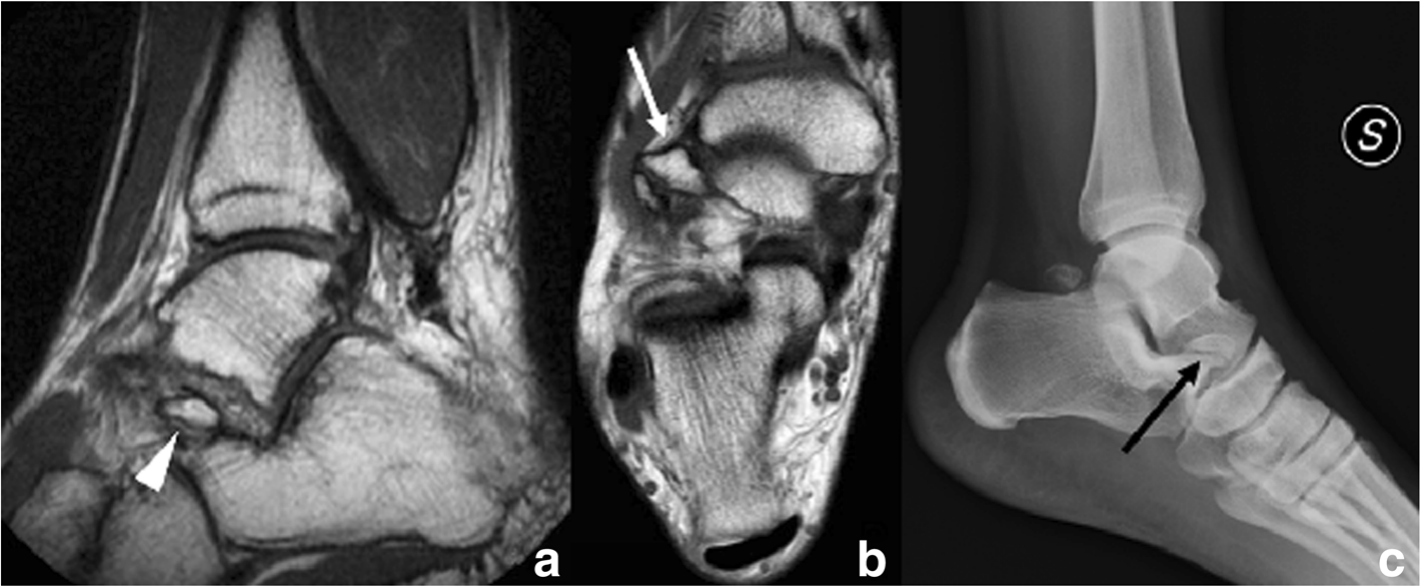
Os calcaneus secundarius. **a** Sagittal FSE T1 in a 56-year-old man referred for follow-up after Achilles reconstructive surgery. As an incidental finding, an os calcaneum secundarium was noted. This is located in between the talus and the calcaneus (white arrowhead). **b** Axial FSE T1 demonstrates the ossicle located in the space between the talus and calcaneus, articulating with the anterior process of the calcaneus (only partially seen) and the tarsal navicular (white arrow). **c** Different patient, incidental finding of an os calcaneus secundarius, here visible on lateral radiographs (black arrow). Note also the presence of an os trigonum

The os calcaneus secundarius is a rare accessory ossicle of the foot, estimated to have a prevalence between 0.6 and 7% [1, 3]. It can be round but is more often triangular in shape and is located in the space in between the anteromedial aspect of the calcaneus, the cuboid, the talar head and the tarsal navicular (Fig. 6) [8].

This normally represents an incidental finding, which can easily be missed on conventional AP and lateral radiographic projections. Oblique views will demonstrate its presence.

The os calcaneus secundarius is sometimes very difficult to distinguish from a fracture of the anterosuperior calcaneal process [18], which usually occurs as an avulsion injury of the bifurcate ligament on forced plantar flexion, but can also happen in eversion injuries with a dorsiflexed foot [19]. Just as the ossicle, these fractures are easy to miss on conventional radiographic projections and better demonstrated on oblique views. Clinically, they manifest with tenderness in this location, which lies anterior and inferior to the anterior talofibular ligament. Due to the location of pain, the fracture can mimic a sprain, a fracture of the lateral talus or the base of the fifth metatarsal [20].

**Fig. 7:**
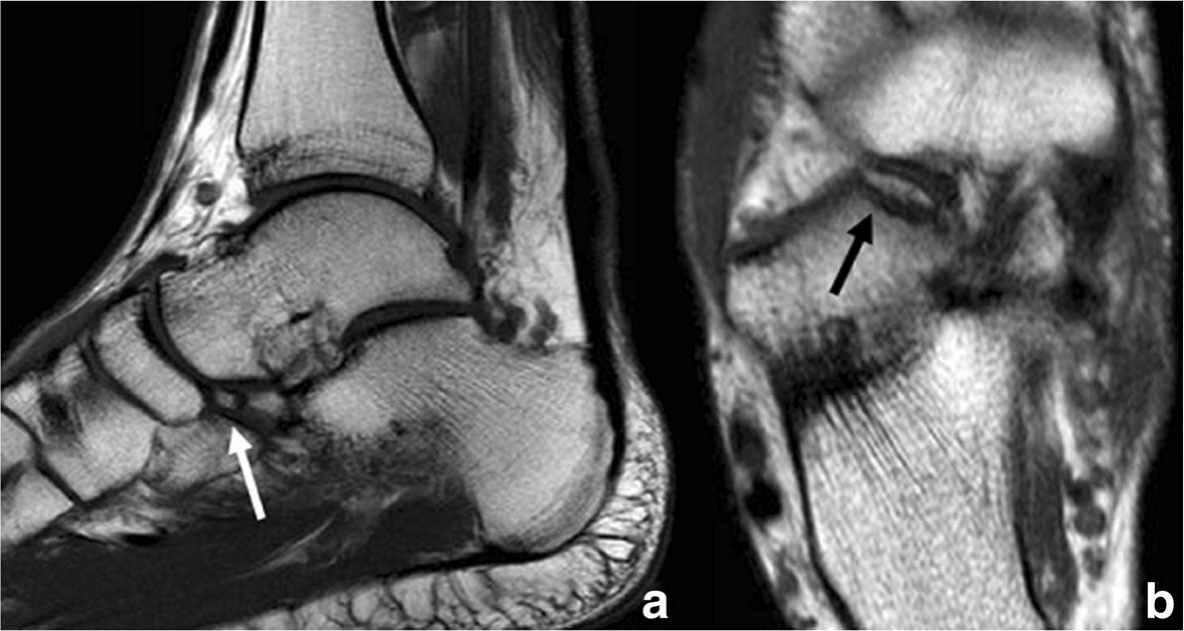
Differential diagnosis of os calcaneus secundarius. 44-year-old man referred with the suspicion of Achilles tendinopathy. **a** Sagittal FSE T1 incidentally demonstrates a small ossified body present in the typical location of an os calcaneus secundarius (white arrow). Patient cannot recall trauma or pain in the lateral aspect of the foot. In these cases, it is occasionally very difficult to establish the diagnosis and distinguish a sequel of an old fracture from a true small ossicle. **b** On axial FSE T1, the bone fragment is elongated and has irregular margins (black arrow). It corresponds to a defect in contour of the calcaneus. This orientates towards a sequel of old fracture as opposed to an accessory ossicle

If one of these fractures is suspected, CT or MRI should be performed to fully characterise, given the implications for treatment [21] (Fig. 7).

Just as with the previously seen ossicles, the diagnosis can be established with the clinical background, and the typical irregularity of margins of fracture and no cortication described [22]. The presence of oedema on MRI confirms the presence of acute fracture [21]. Lack of clinical symptoms and history of trauma and lack of a donor site in the calcaneus are two important features that would suggest the presence of a calcaneus secundarius [23].

### Os subtibiale

The os subtibiale is rare, with an estimated prevalence of 0.9% [2], and is located distal to the tip of the medial malleolus. It can be multiple and bilateral and is usually asymptomatic [24].

The true os subtibiale derives from a persisting accessory centre of ossification and is different from an unfused secondary ossification centre. Secondary ossification centres normally fuse at around 7 years of age. If they do not assimilate to the tibial epiphysis, they appear as a separated medial malleolus [25]. This is more commonly seen than true ossicles.

The main differential of an os tibiale is avulsion fractures, which are common in the context of ankle trauma. Because ankle trauma is the most common indication for radiological examination of the ankle, the accessory ossicle can be mistaken by a fracture [26].

Avulsion fractures are so much more common than the presence of an os subtibiale that in the context of a symptomatic patient after ankle trauma, the finding of an osseous structure below the medial malleolus should be considered and treated as a fracture [27].

### Os subfibulare

The os subfibulare has an estimated prevalence of 2.1% [1, 3]. Similar to the os subtibiale, this represents persistence of an accessory ossification centre, as opposed to an unfused secondary ossification centre, which is more commonly found.

The os subfibulare can be found distal to the tip of the lateral malleolus, with round or comma-shaped morphology, and is asymptomatic [25].

**Fig. 8:**
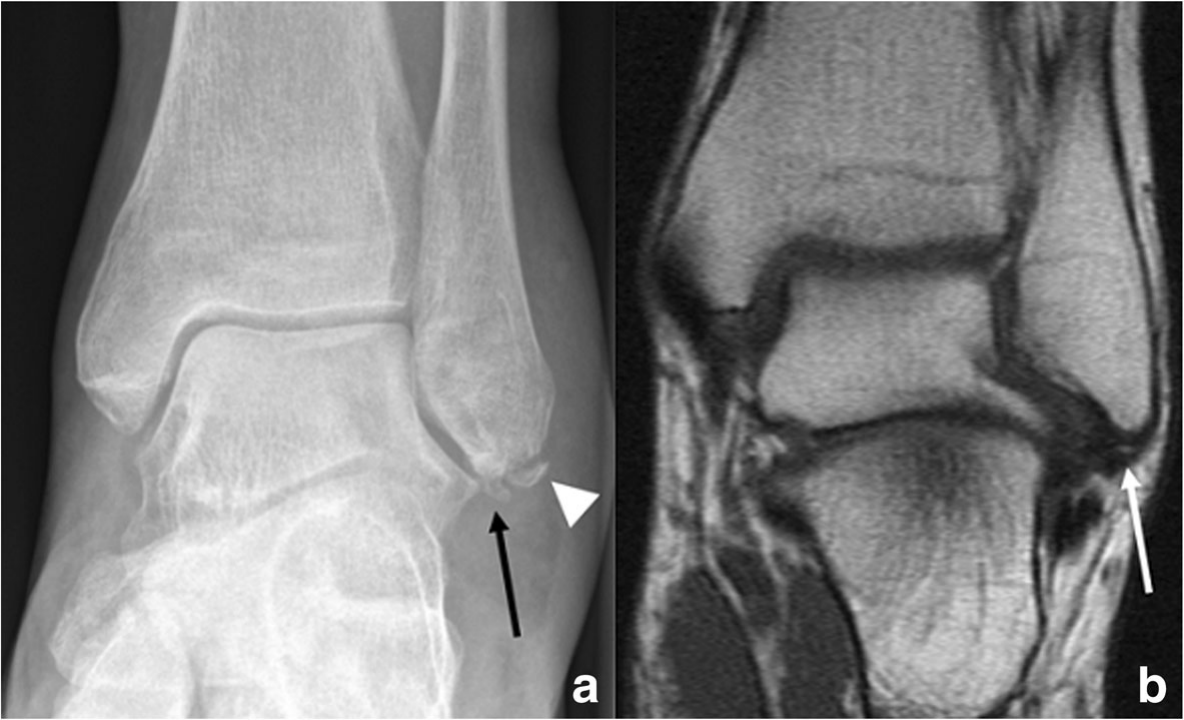
Differential diagnosis of os fibulare. **a** AP radiograph in a 67-year-old woman, history of inversion injury. There is a small avulsion of the tip of the malleolus, in keeping with injury to the lateral collateral ligament (arrow head). Medial to this, there is a small ossified body (black arrow). This has rounded margins. Findings are compared with previous MR, performed 10 years before. **b** Coronal FSE T1 demonstrates a small ossified body was present already (white arrow). MR was performed with the suspicion of talonavicular osteoarthritis at the time. A small rounded structure could have already represented a sequel of avulsion injury but was described as an ossicle in the absence of acute trauma

The main differential is avulsion fractures of the distal fibula. These are typical of inversion injuries of the ankle. Clinically, in these cases, there will be swelling, effusion and pain. Instability may be present, which can be a reliable tool to confirm avulsion fracture, and rule out os subfibulare. Avulsion fractures are more typical of older subjects and normally involve the insertion of the anterior talofibular ligament (Fig. 8). In a lot of cases, the avulsed fragment can have rounded margins, which does not help with differentiation [28].

Given the much higher prevalence of distal fibular injuries due to inversion trauma, some authors actually blanket any ossified structure adjacent to the lateral malleolus as the result of an avulsion fracture, which might have happened remotely in time [2, 29].

### Rare accessory bones

**Fig. 9:**
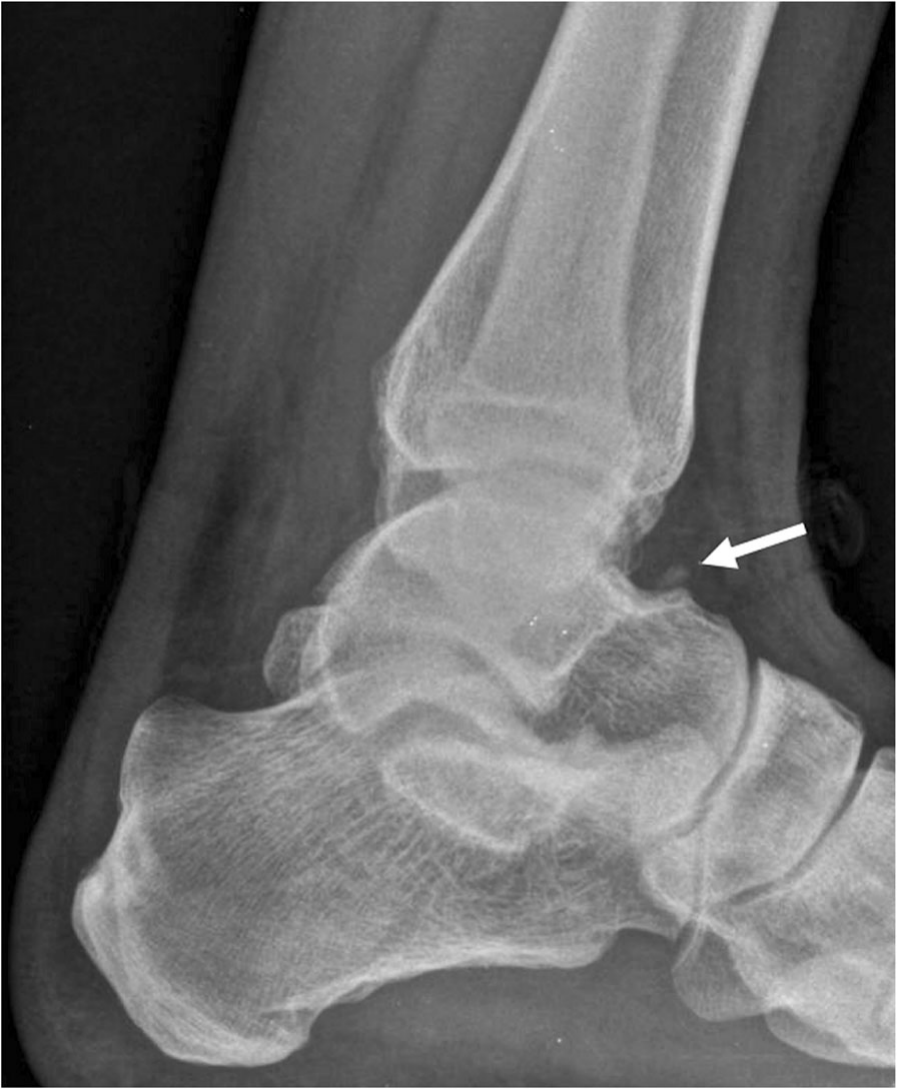
40-year-old man, incidental finding of a small os supratalare (white arrow), as well as an os trigonum. Patient was referred for radiographs with the suspicion of Achilles tendinopathy in the contralateral leg

Other ossicles, such as the os supratalare or os talotibiale, are rare and not associated to painful conditions. The main differential in these cases has to be done with a fracture. Lack of history of trauma, clinical symptoms and absence of a potential site of origin for a fracture fragment in the adjacent bones would favour the existence of an ossicle [8] (Fig. 9).

Several rare accessory bones in the hindfoot have been described, such as an accessory calcaneus, by Krause and Rouse [30], and bipartite configurations of the talus that can be mistaken by fractures [31–33].

Accessory tali are extremely rare, with only a few case reports [34]. These are lateral to the talus. Accessory tali have also been described in association with a partial duplication of the medial column of the foot, in the reported case causing fixed pes equinus deformity [35].

Other rare ossicles have been described, such as an “ankle patella”, a large accessory bone anterior to the tibiotalar joint [36] and a small ossicle located postero-medially to the talus, resulting in tarsal tunnel syndrome [37].

b. Soft tissues of the ankle and hindfoot. Accessory Muscles (Table 2).

### Lateral aspect

#### Accessory peroneal muscles

The peroneal muscles are two, the peroneus longus and peroneus brevis.

They run posteriorly to the lateral malleolus, the brevis more anterior than the longus.

A third peroneus can be found with a prevalence of up to 95% in cadaveric studies [38]. The muscle and tendon are located in the anterior compartment of the leg, arising from the anterior aspect of the distal fibula and the extensor digitorum longus muscle. The tendon normally runs along the extensor digitorum longus tendon and inserts on the dorsal surface of the shaft of the fifth metatarsal. It is normally asymptomatic but has also been described to cause snapping over the lateral dome of the talus [39].

**Table 2:** Prevalence, origin, course, insertion and clinical significance of the accessory muscles around the ankle and hindfoot

	Muscles	Prevalence	Origin	Course	Insertion		Clinical significance
Lateral aspect	Accessory peroneus muscles (peroneus quartus)	16%	Variable Peroneus brevis (most common) Peroneus longus Fibula	Tendon is medial and posterior to the brevis and longus peroneal tendons	Peroneus accessorius	Peroneus longus	Potential crowding in the retinaculum, leading to subluxation of the peroneal tendons or tears due to friction Imaging pitfall—mistaken by tear
					Peroneocalcaneus externum	Calcaneus (79–91%)	
					Peroneus digiti minimis	Head of the fifth metatarsal and base of the first phalanx	
Posteromedial aspect	Flexor digitorum accessorius longus	6–8%	Medial margin of the tibia or from the lateral aspect of the fibula distal to the origin of the flexor hallucis longus	Beneath the flexor retinaculum, through the tarsal tunnel, superficial to the neurovascular bundle	Quadratus plantae or flexor digitorum longus		Tarsal tunnel syndrome Flexor hallucis longus tenosynovitis
	Peroneocalcaneus internus	1%	Internal aspect of the fibula, below the origin of the flexor hallucis longus	Posterior to flexor hallucis longus displacing it anteriorly and medially, with the tendons running parallel	Small tubercle on the medial aspect of the calcaneus, below the sustentaculum		Crowding in the tarsal tunnel Occasionally, limitation of movement, posterior ankle impingement and flexor hallucis longus tenosynovitis
	Accessory soleus	0.7–5.5%	Anterior surface of the soleus, partially sharing the soleus origin	Antero-medially to the Achilles, superficial to the flexor retinaculum	Achilles tendon		Soft tissue mass (incidental finding) Associated pain, triggered by exercise Tarsal tunnel syndrome
	Tibiocalcaneus internus	–	Medial tibia	Deep in the flexor retinaculum	Medial calcaneus		Tarsal tunnel syndrome
Anterior aspect	Peroneus tertius	95%	Anterior aspect of the distal fibula and the extensor digitorum longus muscle	Tendon normally running along the extensor digitorum longus tendon	Dorsal surface of the shaft of the fifth metatarsal		normally asymptomatic, however snapping of its tendon over the lateral dome of the talus has been described
	Extensor hallucis capsularis tendon	14%	Extensor hallucis longus tendon or muscle	Parallel extensor hallucis longus	First metatarsophalangeal joint capsule		Grafting if needed for reconstruction, especially in cases of hallux dysfunction
	Anterior fibulocalcaneus	Rare	Fibula, peroneus tertius	Tendon parallel to the extensors	Calcaneus, anterosuperior to fibular throclea		Pain due to impingement
	Accessory extensor digiti secundus	Rare	Extensor hallucis longus tendon or muscle	Tendons parallel to the extensors	Medial phalanx second digit		Incidental finding
	Tibioastragalus anticus of Gruber	Rare	Lower third of the anterolateral tibia and interosseous membrane	Tendon is deep to the tibialis anterior and extensor hallucis longus tendon	Anterolateral aspect of the neck of the talus		Potential tendon transfer

##### Peroneus quartus

There are a number of other accessory peroneal muscles, with names such as peroneus accessorius, peroneocalcaneus externum, peroneus digiti minimis and peroneus quartus. This last term is the one generally used for accessory peroneal muscles in the postero-lateral aspect of the leg [40].

Prevalence is difficult to set. In general, cadaveric and radiological studies demonstrate similar prevalence, approximately 10% on MRI [41] and 22% on ultrasound [42]. A recent review of 46 studies reported an overall prevalence of 16% [43].

It is more commonly bilateral and seen in males.

In most cases, the peroneus quartus arises from the peroneus brevis, but it can arise from the peroneus longus and fibula as well. The tendon is medial and posterior to the brevis and longus peroneal tendons.

The insertion is very variable and will determine the different given names. The most common insertion is on the calcaneus, known as peroneocalcaneus externum, with a prevalence of 79–91% [44].

**Fig. 10:**
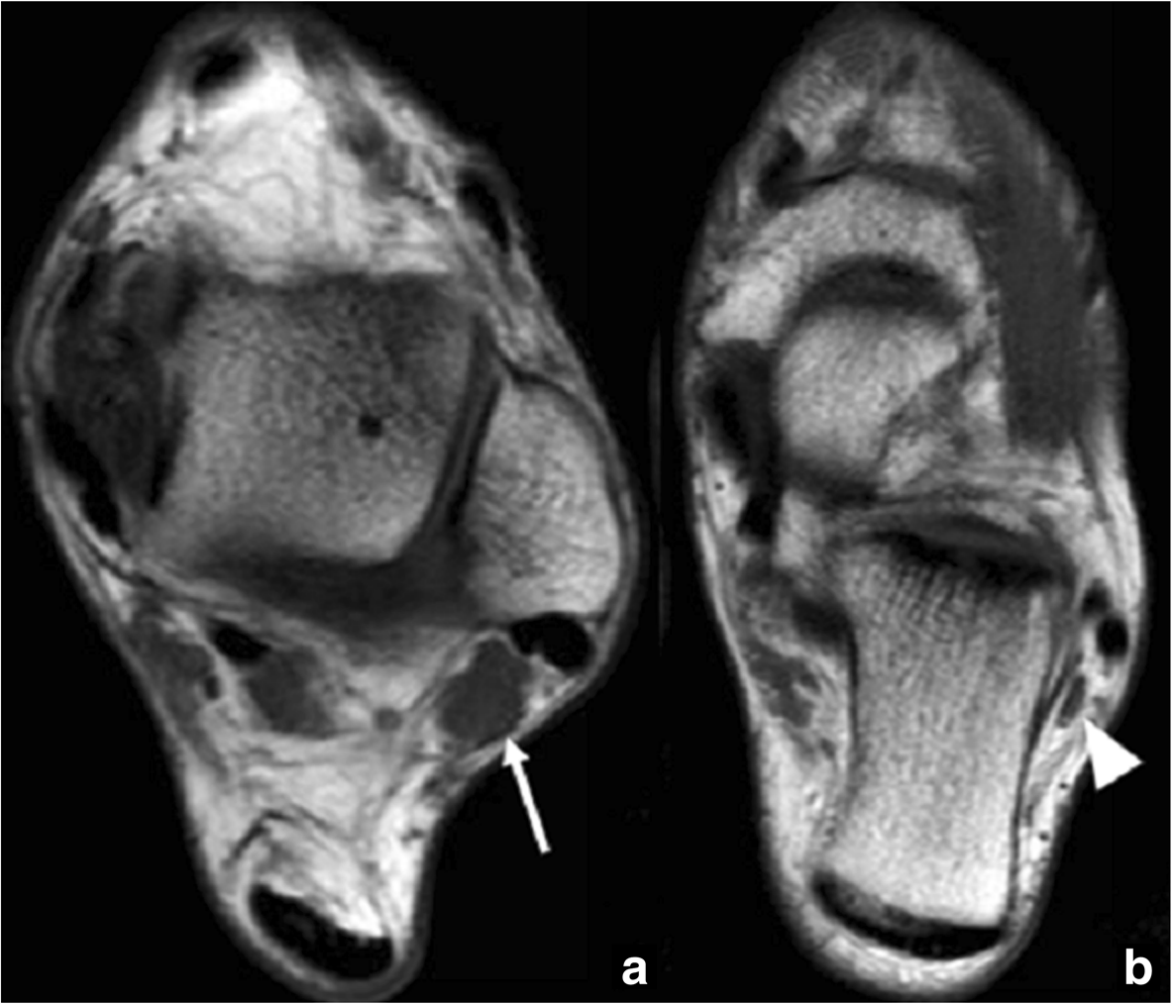
Peroneus quartus. 44-year-old man referred for follow-up of an osteochondral lesion in the talus. Axial FSE T1 images in different planes (**a**) proximal and (**b**) distal demonstrate the incidental finding of a peroneus quartus (white arrow). This descends to insert in the lateral aspect of the calcaneus, with a fleshy attachment (arrowhead)

The peroneus accessorius arises from the peroneus brevis to insert onto the peroneus longus, and the peroneus digiti minimi arises from the peroneus brevis and inserts onto the head of the fifth metatarsal and base of the first phalanx [38, 40]. Insertion on the peroneus brevis, cuboid and peroneus longus or inferior retinaculum have also been described [45] (Fig. 10).

The peroneus quartus is a pronator. It is normally asymptomatic, but in some cases, it can cause crowding in the retinaculum, leading to subluxation of the peroneal tendons or tears due to friction.

A peroneus quartus can be removed in the cases in which it is causing pathology. A very interesting feature of this accessory muscle is that it can be used on ankle surgery to repair retinaculum injuries [38].

Finally, the peroneus quartus can result in imaging pitfalls. The accessory tendon of the peroneus quartus, separated by a tissue plane from the other peroneal tendons, can be mistaken for a tear, but distinguishing it becomes easy by following the tendon to its own independent muscle belly [42].

### Posteromedial aspect

#### Flexor digitorum accessorius longus

The flexor digitorum longus arises from the tibial surface, below the origin of the soleus. The tendon courses posterior to the medial malleolus and splits into four different tendons that insert on the distal phalanxes of the second to fifth toes.

Prevalence of the flexor digitorum accessorius longus has been set in 6–8% [46]. It is more common in males and it is unusual to find it bilaterally.

**Fig. 11:**
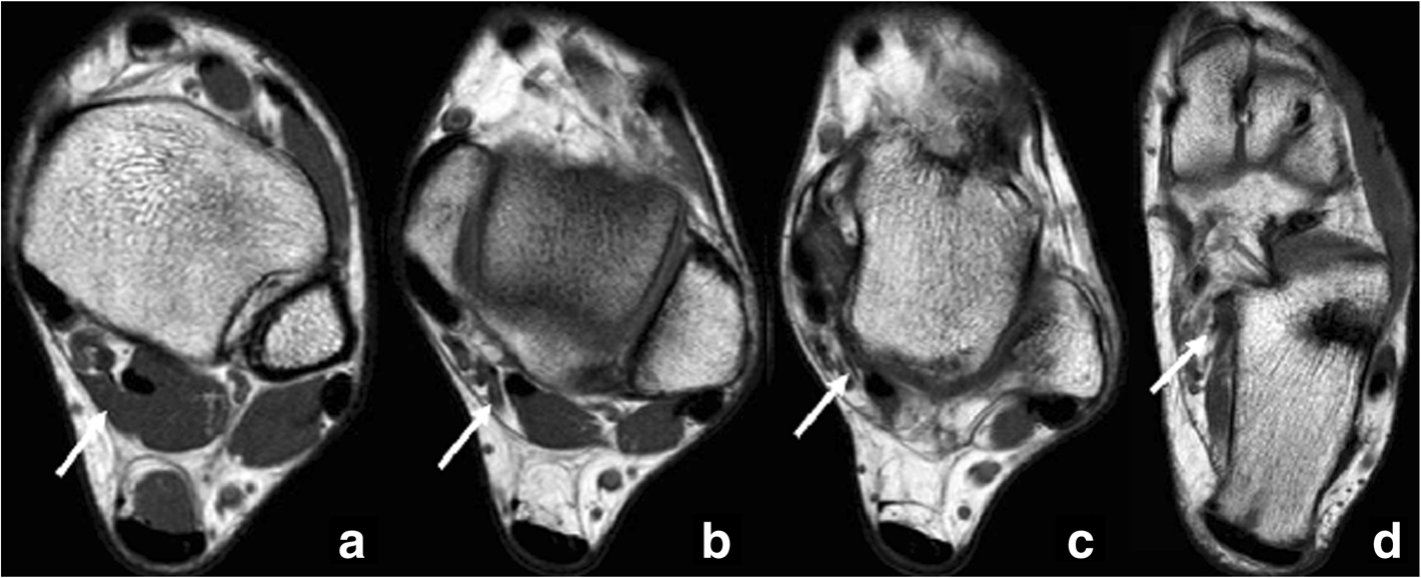
Flexor digitorum accessorius longus inserting in the quadratus plantae. 35-year-old man, referred for pain in the Achilles. **a**-**d** Axial FSE T1 images at different planes from proximal to distal show the course of a flexor digitorum accessorius longus. This is medial to the flexor hallucis longus and is included in the flexor retinaculum. The accessory muscle inserts in the quadratus plantae (white arrows)

The flexor digitorum accessorius longus can arise from any structure in the posterior compartment but is seen to arise more frequently from the medial margin of the tibia or from the lateral aspect of the fibula distal to the origin of the flexor hallucis longus. The tendon courses beneath the flexor retinaculum, through the tarsal tunnel, superficial to the neurovascular bundle (posterior tibial artery and nerve). It typically inserts onto the quadratus plantae or flexor digitorum longus [46] (Fig. 11).

In the tarsal tunnel, the tendon or low-lying fibres of the muscle can create a compromise of space, and so the presence of a flexor digitorum accessorius longus has been linked to tarsal tunnel syndrome [47]. One surgical report found that up to 12% cases of tarsal tunnel were caused by flexor digitorum accessorius longus [44, 48].

The presence of this accessory muscle has also been associated to flexor hallucis longus tenosynovitis [49].

#### Peroneocalcaneus internus

The peroneocalcaneus internus muscle is a rare accessory muscle. In a series of asymptomatic volunteers, its prevalence was estimated on 1% [44]. In one of four cases, this will be a bilateral finding.

**Fig. 12:**
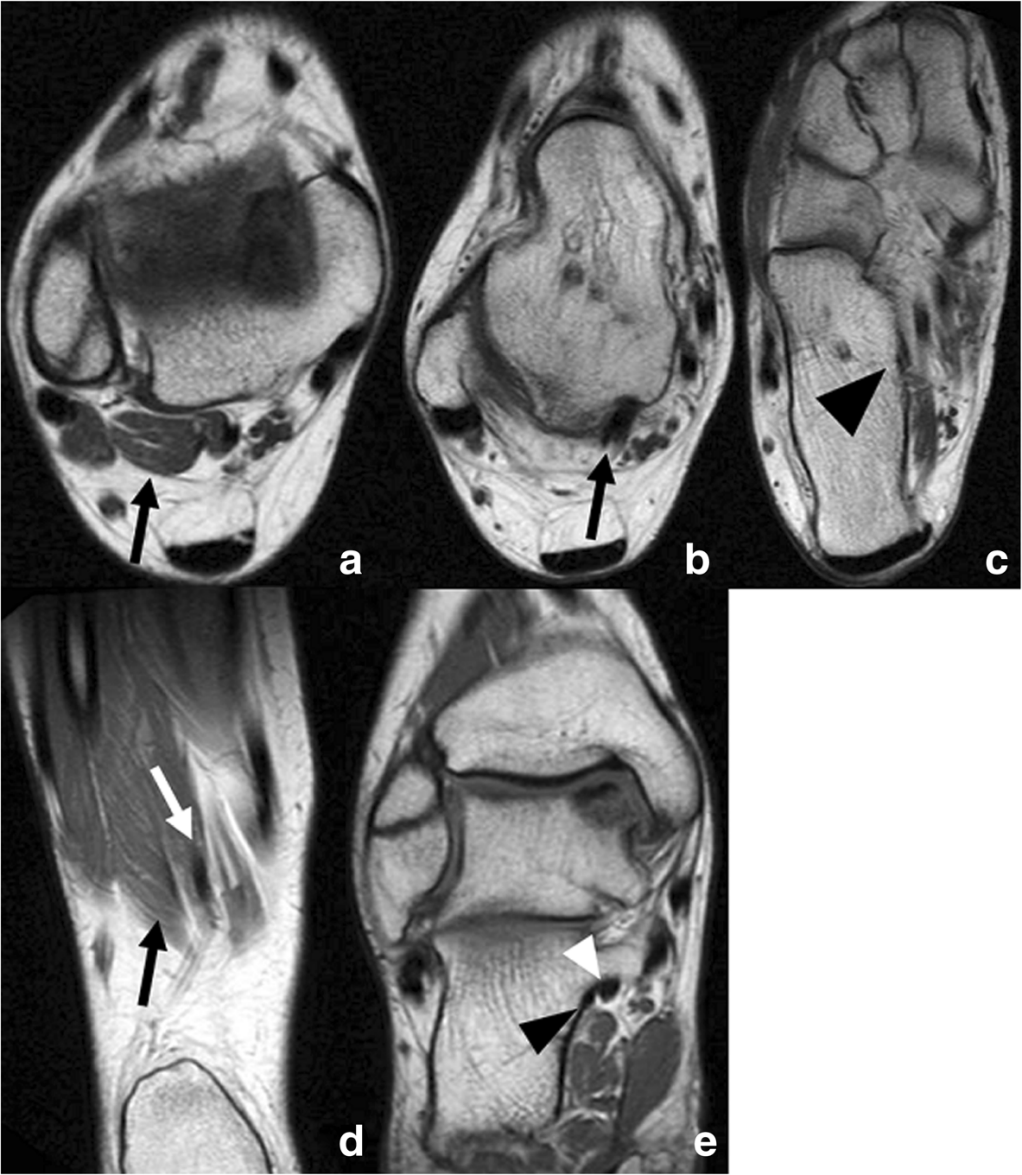
Peroneocalcaneus internus (PCI). 17-year-old woman for follow-up for osteochondral lesion in talus. Incidental finding of a peroneocalcaneus internus. **a**-**c** Axial FSE T1 at different levels from proximal to distal (black arrows) demonstrates the muscle belly and tendon, descending parallel to the flexor hallucis longus and inserting into the medial aspect of the calcaneus (black arrowhead). Coronal FSE T1 in two slices, from (**d**) posterior to (**e**) anterior in the same patient nicely depicts the PCI tendon (black arrow) parallel to the flexor hallucis longus (white arrow), descending to insert into the calcaneus, below the sustentaculum (black arrowhead), more medial than the flexor hallucis longus (white arrowhead)

The peroneocalcaneus arises from the internal aspect of the fibula, below the origin of the flexor hallucis longus, and descends posterior to this displacing it anteriorly and medially, with the tendons running parallel. This configuration has the potential to cause crowding in the tarsal tunnel [50, 51]. The peroneocalcaneus internus inserts on a small tubercle on the medial aspect of the calcaneus, below the sustentaculum (Fig. 12).

The presence of the muscle is normally asymptomatic, given it is not directly related to the neurovascular bundle. Only in very rare cases in which the peroneocalcaneus internus displaces the flexor hallucis longus medially a tarsal tunnel syndrome has been reported [50]. Occasionally, limitation of movement, posterior ankle impingement and flexor hallucis longus tenosynovitis have also been described in association to its presence.

It can be mistaken with a flexor digitorum accessorius longus, but its location posterior to the flexor hallucis longus, as opposed to the neurovascular bundle, and its insertion on the calcaneus, as opposed to the flexor digitorum longus or quadratus plantae, help on distinction [38].

A rare associated problem with the presence of a peroneocalcaneus internus is the possibility to fail to recognise this separately from the flexor hallucis longus in arthroscopy (mistaking it with the flexor hallucis longus tendon), which may lead to an altered surgical approach, and potential injury to the neurovascular bundle. The flexor hallucis longus is used as a landmark for the medial margin of safety in arthroscopic surgery [44].

#### Accessory soleus

The soleus is located in the deep posterior compartment and arises from the proximal fibula, the posterior (soleal line) and medial tibia and a fibrous line bridge in between tibia and fibula. Its tendon normally joins the Achilles tendon.

Accessory solei have a prevalence of 0.7–5.5% according to cadaveric studies [52].

**Fig. 13:**
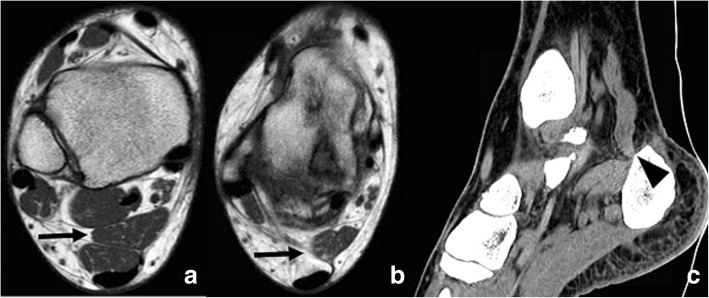
Accessory soleus. 36-year-old woman referred for follow-up of an osteochondral lesion. Incidental finding of an accessory soleus. Axial FSE T1 at different levels from (**a**) proximal to (**b**) distal demonstrates the muscle belly anterior to the soleus, superficial to the flexor retinaculum (black arrows). The tendon descends to insert in the medial aspect of the calcaneus. **c** Sagittal plane CT reconstruction with soft tissue algorithm in the same patient also allows visualisation of the muscle, extending to insert in the calcaneus (black arrowhead)

The accessory soleus normally arises from the anterior surface of the soleus, partially sharing the soleus origin, and descends antero-medially to the Achilles, superficial to the flexor retinaculum, to insert on either the Achilles or the calcaneus, on the superior or medial surface, with a tendinous or a fleshy insertion. The different types of insertion define the different types of classified accessory soleus [53] (Fig. 13).

An accessory soleus may present as a soft tissue mass in the postero-medial aspect of the ankle. It tends to be noted if there is an increase in muscle mass and activity, and sometimes, there is associated pain, triggered by exercise, that could be explained by the increase of intrafascial pressure or insufficient blood supply [52, 53]. In some cases, it may cause compression of the posterior tibial nerve, and associated tarsal tunnel syndrome has been described in cases in which the accessory soleus inserts onto the medial calcaneus.

Accessory solei may be evident on radiographs, as partial obliteration of the Kager fat pad. MRI and CT are more sensitive and specific, and in the case of MRI, intrinsic muscular changes and relations with adjacent structures can be evaluated to investigate the aforementioned associated pathologies [44, 53].

#### Tibiocalcaneus internus

The tibiocalcaneus is a rare accessory muscle, with only a few radiology reports [44, 54].

It has been described arising from the medial tibia and inserting on the medial calcaneus, similarly to a subtype of accessory soleus, with the difference that the tibiocalcaneus is located deep in the flexor retinaculum. As a difference from the flexor digitorum accessorius longus, the tibiocalcaneus inserts onto the calcaneus and not the flexor digitorum longus or quadratus plantae.

Given the deep location in the flexor retinaculum, this accessory muscle can potentially cause tarsal tunnel syndrome [38].

### Anterior aspect

A third peroneus muscle and tendon located in the anterior compartment is a common finding that can be found with a prevalence of up to 95% in cadaveric studies [38]. The muscle arises from the anterior aspect of the distal fibula and the extensor digitorum longus muscle, with the tendon normally running along the extensor digitorum longus tendon and inserting on the dorsal surface of the shaft of the fifth metatarsal. The presence of the third peroneus is normally asymptomatic; however, snapping of its tendon over the lateral dome of the talus has been described [39].

The extensor hallucis capsularis tendon has been estimated to have a prevalence of 14% in the population. The tendon arises from the extensor hallucis longus tendon or muscle, in most of the cases, and inserts onto the first metatarsophalangeal joint capsule. This tendon may be useful in the cases in which grafting is needed for reconstruction, especially in cases of hallux dysfunction [55].

Other rare accessory muscles have been described in the anterior compartment of the leg, with tendons parallel to the extensors, such as the anterior fibulocalcaneus [56], which originates in the fibula and peroneus tertius and inserts in the calcaneus, potentially causing pain due to impingement, and a variation of the extensor hallucis longus, as an accessory extensor digiti secundus [57, 58], which originates with the extensor hallucis longus, runs parallel to it and inserts in medial phalanx of the second digit. This is normally an incidental finding, with no clinical implication.

The tibioastragalus anticus of Gruber’s muscle is another rare accessory muscle in the anterior compartment of the leg. The muscle arises from the lower third of the anterolateral tibia and interosseous membrane and inserts onto the anterolateral aspect of the neck of the talus. The tendon is deep to the tibialis anterior and extensor hallucis longus tendon [59]. This accessory muscle can be used for tendon transfer or graft.

## Conclusion

This review has illustrated the imaging findings related to the presence of accessory ossicles and muscles in the ankle and foot through different techniques and the potential clinical implications related to their existence, highlighting the importance of each technique in the diagnosis and assessment of related pathology.

Most accessory ossicles will represent an incidental finding on radiographs. Accessory muscles can occasionally represent an incidental finding on radiographs, but are mainly incidentally noted on MRI and CT.

In cases where pathology in relation to the presence of these structures is suspected, detailed clinical correlation and careful assessment with MRI and CT plays a very important role.

It is useful for the radiologist to be familiar with the characteristics of these anatomical variants to avoid misdiagnosis.

## Data Availability

Not applicable

## Abbreviations

CT: Computed tomography
FFE: T2-weighted fast field echo
FSE PD: Fast spin-echo proton density
FSE T1: Fast spin-echo T1
MRI: Magnetic resonance imaging
PD SPAIR: Proton density spectral attenuation inversion recovery
US: Ultrasound
